# Central blood pressure and measures of early vascular disease in children with ADPKD

**DOI:** 10.1007/s00467-019-04287-7

**Published:** 2019-06-26

**Authors:** Matko Marlais, Sreedevi Rajalingam, Haotian Gu, Alexandra Savis, Manish D Sinha, Paul JD Winyard

**Affiliations:** 1 UCL Great Ormond Street Institute of Child Health, 30 Guilford St, Holborn, London, WC1N 1EH UK; 2 Department of Paediatric Nephrology, Evelina London Children’s Hospital, Westminster Bridge Rd, Lambeth, London, SE1 7EH UK; 3 Department of Paediatric Cardiology, Evelina London Children’s Hospital, Guy’s & ST Thomas’ Foundation Hospitals NHS Trust, Westminster Bridge Road, London, SE1 7EH UK; 4 Kings College London, London, UK

**Keywords:** Children, Polycystic kidney disease, Cardiovascular

## Abstract

**Background:**

There is growing recognition of hypertension in a significant proportion of children with ADPKD. In this study, we assessed blood pressure and cardiovascular status in children with ADPKD.

**Methods:**

A prospective two-centre observational study of children (< 18 years) with ADPKD was compared against age- and BMI-matched healthy controls. Children underwent peripheral BP (pBP) measured using an aneroid sphygmomanometer and auscultation, 24-h ambulatory BP monitoring (ABPM), non-invasive central BP (cBP) measurement, carotid-femoral pulse wave velocity (PWVcf) measured using applanation tonometry and measurement of indexed left ventricular mass (LVMI) using echocardiography. This study received independent ethical approval.

**Results:**

Forty-seven children with ADPKD and 49 healthy controls were recruited (median age 11 years vs. 12 years). Children with ADPKD had significantly higher systolic pBP (mean 112 ± 13.5 mmHg vs. 104 ± 11 mmHg, *p* < 0.001), higher systolic cBP (mean 97 ± 12.8 mmHg vs. 87 ± 9.8 mmHg, *p* < 0.001) and lower pulse pressure amplification ratio (1.59 ± 0.2 vs. 1.67 ± 0.1, *p* = 0.04) compared to healthy children. Thirty-five percent of children with ADPKD showed a lack of appropriate nocturnal dipping on 24-h ABPM. There was no difference in PWVcf between children with ADPKD and healthy children (mean 5.74 ± 1 m/s vs. 5.57 ± 0.9 m/s, *p* = 0.46). Those with ADPKD had a significantly higher LVMI (mean 30.4 ± 6.6 g/m^2.7^ vs. 26.2 ± 6.2 g/m^2.7^, *p* = 0.01).

**Conclusions:**

These data highlight the high prevalence of hypertension in children with ADPKD, also demonstrating early cardiovascular dysfunction with increased LVMI and reduced PP amplification despite preserved PWVcf, when compared with healthy peers. These early cardiovascular abnormalities are likely to be amenable to antihypertensive therapy, reinforcing the need for routine screening of children with ADPKD.

## Introduction

Autosomal dominant polycystic kidney disease (ADPKD) was traditionally considered an ‘adult’ disease [1], but recent evidence from multiple studies has shown a significant prevalence of hypertension and other features suggestive of early cardiac damage during childhood [2–4]. Despite this, most children at risk of ADPKD do not receive regular or consistent medical follow-up [5, 6]. There are still no guidelines recommending routine diagnostic testing of children with ADPKD; rather children at risk (by virtue of their family history) can be monitored and then evaluated if any problems are detected, until direct anti-ADPKD therapies gain approval in children [7].

The commonest significant clinical issue in ADPKD during childhood appears to be hypertension, which we reported at ~ 20% of affected children in a recent meta-analysis [4]. Some studies (including the largest single study published to date) report higher prevalence of hypertension in children with ADPKD, up to 35% [8]. It should be recognised, however, that many of these studies come from specialist clinics which make them susceptible to selection bias; current estimates may therefore overestimate the prevalence of hypertension in this population. It is perhaps paradoxical that adult studies report median age at diagnosis of hypertension as 33 years with a very low number diagnosed before age 20; it is likely these figures reflect lack of screening in those with ADPKD in the absence of overt symptoms [9].

The mechanism underlying childhood hypertension in ADPKD remains unknown, and it precedes the decline in renal function. However, the strongest evidence exists for progressive cyst expansion and proliferation affecting regional perfusion and drainage, leading to activation of the renin-angiotensin-aldosterone system [10, 11]. This leads to vasoconstriction with salt and water accumulation. Other potential mechanisms include endothelial dysfunction, increased vasopressin levels and sympathetic overactivity [10].

Although hypertension in children with ADPKD is now increasingly recognised, literature on other aspects of their cardiovascular status remains limited. Increased left ventricular mass index (LVMI) in children with ADPKD who are hypertensive has been reported, but interestingly, this study also found an increased LVMI in those with borderline hypertension [3]. To our knowledge, there have only been two previous studies considering other measures of vascular health in children with ADPKD. These reported early vascular disease, but the studies were small (*n* = 36 in total), included young adults above 18 years of age and had only 23 children (64%) aged 16 or younger across both studies combined [12, 13].

Recent adult evidence highlights markers of vascular health in predicting adverse cardiovascular outcomes. In particular, central blood pressure (cBP) appears more closely linked to cardiovascular risk than peripheral blood pressure (pBP) [14]. Along with the studies mentioned above [12, 13], cBP has previously been studied in 18 adults (mean age 34 years) with ADPKD and they found evidence of early arterial pathology in this population [15].

The primary aim of this study was to assess vascular health in children with ADPKD by measuring pBP and cBP, carotid-femoral pulse wave velocity (PWVcf) and left ventricular mass index (LVMI) and by comparing these to age- and BMI-matched healthy controls. Secondary aims included comparing pBP values with 24-h ambulatory BP monitoring (ABPM) in those with ADPKD, as well as assessment of microalbuminuria and estimated glomerular filtration rate.

## Materials and methods

### Participants

This was a prospective two-centre observational study conducted across two large paediatric nephrology units in London, UK: Evelina London Children’s Hospital (ELCH) and Great Ormond Street Hospital for Children. Children and adolescents (< 18 years) were consecutively recruited from paediatric nephrology clinics and a dedicated polycystic kidney disease clinic at ELCH. Inclusion criteria were a confirmed diagnosis of ADPKD (confirmed through ultrasonography using the Modified Ravine criteria, or in those aged under 15 years, a finding of renal cysts in the context of a family history of ADPKD—criteria used in the recent large pan-European study) [8]. Exclusion criteria were those children with unilateral cysts in the absence of a family history of ADPKD. We also excluded children aged under 3, as they were felt to be unlikely to tolerate detailed vascular assessments. Comparisons in this study were made against a group of age- and BMI-matched healthy controls, who were recruited from the local population. Formal ethical approval for this study was granted following external ethical review by the Hampstead Research Ethics Committee at Royal Free Hospital, London (REC number 15/LO/1100). All participants in this study (or their parents) gave written informed consent, plus children gave their assent where age-appropriate.

Baseline data was collected for all those studied with ADPKD, including demographics, latest renal ultrasound findings, method of confirmation of ADPKD and presence of a family history. We also collected data on current or historical symptoms and signs related to ADPKD, including those with pre-existing hypertension (defined as a systolic or diastolic BP above the 95th percentile for age, sex and height or the use of antihypertensive medication). We used definitions as per the 2016 European Society of Hypertension Guidelines [16]. In those with ADPKD, we recorded the random urine albumin to creatinine ratio; microalbuminuria was defined as a urine albumin to creatinine ratio above the normal range (0–2.4 g/mol). The urine was collected in a clinic setting and was not always the first morning urine. We calculated their estimated glomerular filtration rate (eGFR) using the New Modified Schwartz formula from 2009 [17, 18]. Demographic data was collected for healthy controls, as well as eGFR if blood tests were done, but formal laboratory urine testing was not performed.

### Blood pressure measurement

Peripheral systolic and diastolic BP was measured in participants seated for 5 min in a quiet environment. Trained observers measured peripheral BP at the brachial artery thrice by auscultation using a calibrated aneroid sphygmomanometer, and the mean was used for further analyses. *Z*-scores for pBP were calculated according to the Fourth Report [19].

Radial artery applanation tonometry was performed next on the right wrist of each subject by lightly applying a high-fidelity micromanometer (SPC-301; Millar Instruments, Houston, TX) to the radial artery. All readings were processed by the SphygmoCor device (AtCor Medical, West Ryde, New South Wales, Australia), which gave an estimate of cBP through calibration with pBP. Only those radial waveforms meeting the inbuilt quality control criteria of the SphygmoCor device were accepted. Three estimates of cBP were taken, and the mean of these readings was recorded. Pulse pressure (PP) amplification was assessed as the ratio of peripheral to central pulse pressure (brachial to aortic PP).

Twenty-four-hour ABPM was performed in children with ADPKD using the Spacelabs Healthcare 90217 recorder (OSI Systems, Hawthorne, California) and an appropriately sized cuff; children too young to tolerate the ABPM were excluded. Mean 24-h, daytime and nocturnal BP values were recorded. An ABPM study was excluded if (1) there was 2 h of interrupted recordings or (2) the duration of recording was inadequate (i.e. 20 h in total, 12 h continuously or did not include night-time measurements) [20]. Twenty-four-hour ABPM data was compared to clinic pBP data as above.

### Cardiovascular assessments

In addition to cBP measurement and pulse pressure amplification as above, further assessment of vascular health was undertaken with carotid-femoral pulse wave velocity (PWVcf) measurement. Arterial waveforms were obtained from the carotid and femoral arteries with the patient in the supine position; these were processed using the SphygmoCor device, and PWVcf was calculated with simultaneous 3-lead ECG monitoring. PWVcf was measured three times in succession, and the mean value was recorded. PWVcf *Z*-scores were calculated using the LMS method with data from Reusz et al. [21].

Indexed left ventricular mass (LVMI) was measured using standard two-dimensional m-mode echocardiography. All echocardiograms were performed by a single trained operator who was blinded to the diagnosis, blood pressure and other vascular data. Left ventricular hypertrophy was defined as LVMI > 51 g/m^2.7^.

### Statistical analyses

Summary statistics are presented as means for continuous data and proportions for categorical data. Comparisons between the two groups in this study were tested using the dependent or independent sample *t* test for continuous data if the data were normally distributed (checked using the Shapiro-Wilk test). Where data were not normally distributed, the non-parametric Mann-Whitney *U* test was used for independent samples. The chi-squared test was used for categorical data. Statistical analyses were performed using SPSS version 22 (SPSS Inc., Chicago, Illinois). All tests were two-tailed, and a *p* value < 0.05 was taken to represent a statistically significant result.

## Results

Forty-seven children with ADPKD and 49 healthy controls were recruited with groups well matched for age and BMI (Table 1). The median age of those with ADPKD was 11 years, and two children were under the age of 5 years at the time of the study. There was a significantly lower eGFR in the ADPKD group when compared with healthy controls, but most were normal and only three children (6%) with ADPKD had an eGFR < 60 mL/min/1.73 m^2^.

**Table 1:** Baseline characteristics

	ADPKD (*n* = 47)	Healthy (*n* = 49)	*p* value
Median age (range), years	11 (3–17)	12 (6–17)	0.91
Male	26 (55%)	19 (39%)	0.10
Median weight (range), kg	42 (16–120)	48 (22–107)	0.25
Median height (range), cm	152 (103–189)	153 (125–186)	0.62
Median BMI (range), kg/m^2^	18 (15–34)	20 (14–38)	0.17
Median eGFR (range), mL/min/1.73 m^2^	86 (49–147)	103 (71–120)	0.004

Missing data: 4 children in the ADPKD group and 15 children in the healthy control group did not have blood tests done, and therefore, eGFR was not estimated

*BMI* body mass index, *eGFR* estimated glomerular filtration rate

Table 2 shows the clinical characteristics of the ADPKD group; in total, 10 children (21%) met the criteria for hypertension in this study. Interestingly, 5 children (11%) were prenatally diagnosed or suspected to have ADPKD; one of these children was known to have inherited a PKD1 mutation from one parent and a hypomorphic mutation in the other. Twelve children with ADPKD (26%) in this study had history of recurrent urinary tract infections (UTIs). Eighteen children with ADPKD (out of 43 where urine testing was completed, 42%) had microalbuminuria, with the mean albumin to creatinine ratio on a study level only slightly higher than the upper limit of the normal range.

**Table 2:** Clinical characteristics in 47 children with ADPKD

Blood pressure	On antihypertensive treatment	8 (17%)
	Hypertensive but not on treatment	2 (4%)
Clinical characteristics and symptoms	Family history of ADPKD	43 (91%)
	Antenatally diagnosed/suspected	5 (11%)
	Abdominal/back pain	5 (11%)
	Recurrent UTIs	12 (26%)
Assessment of proteinuria	Raised random albumin to creatinine ratio	18 (42%)
	Mean albumin to creatinine ratio (g/mol)	4.6

Missing data: 4 children in the ADPKD group did not have urine tests

*UTI* urinary tract infection

### Blood pressure

**Fig. 1:**
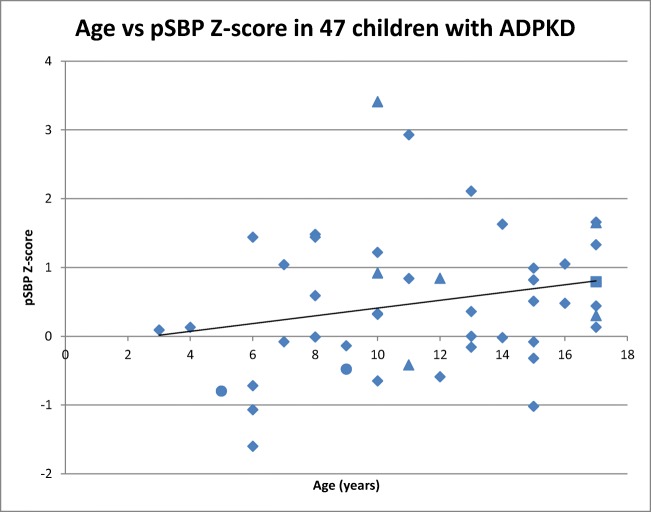
Graph showing peripheral systolic blood pressure *Z*-score against age in 47 children with ADPKD. *R*^2^ = 0.0519 (*p* = 0.12). Marker coding: triangle, children with ADPKD who were on anti-hypertensive medication at the time of the study; circle, children with ADPKD who were both on anti-hypertensive medication at the time of the study and had an eGFR < 60 mL/min/1.73 m^2^; square, child with ADPKD who had an eGFR < 60 mL/min/1.73 m^2^; diamond, all other children with ADPKD in the study

Children with ADPKD had significantly higher peripheral and central blood pressure levels (Table 3). We found a significantly lower PP amplification ratio in the ADPKD group, compared to the healthy controls, suggesting that there is a degree of arterial stiffening in this population. There was a statistically significant positive correlation between the PP amplification ratio and age (*r*_s_ 0.41, *p* = 0.01) and a negative correlation with eGFR (*r*_s_ − 0.47, *p* = 0.005) Spearman rank correlation coefficient. Figure 1 shows the pSBP *Z*-score plotted against age in 47 children with ADPKD, showing a tendency towards a higher blood pressure *Z*-score with increasing years, but this was not statistically significant (*R*^2^ = 0.0519, *p* = 0.12). This figure highlights those who were on anti-hypertensive medication at the time of the study and those with a reduced eGFR.

**Table 3:** Blood pressure and cardiovascular assessments

	ADPKD (*n* = 47)	Healthy (*n* = 49)	*p* value
Mean (SD) pSBP (mmHg)	112 (± 13.5)	104 (± 11)	< 0.001
Mean (SD) pSBP *Z*-score	0.49 (± 1)	− 0.33 (± 0.8)	< 0.001
Mean (SD) pDBP (mmHg)	65 (± 12.1)	60 (± 12.8)	0.04
Mean (SD) pDBP *Z*-score	0.27 (± 1)	− 0.26 (± 1.1)	0.01
Mean (SD) cSBP (mmHg)	97 (± 12.8)	87 (± 9.8)	< 0.001
Mean (SD) cDBP (mmHg)	67 (± 12.1)	61 (± 12.2)	0.04
Mean (SD) peripheral PP (mmHg)	47 (± 9.7)	43 (± 12.5)	0.15
Mean (SD) central PP (mmHg)	30 (± 5.3)	26 (± 7.7)	0.006
Mean (SD) ratio peripheral/central PP	1.59 (± 0.2)	1.67 (± 0.1)	0.04
Mean (SD) PWVcf (m/s)	5.74 (± 1)	5.57 (± 0.9)	0.46
Mean (SD) PWVcf *Z*-score	1.21 (± 1)	0.78 (± 0.9)	0.07
Mean (SD) echo LVMI (g/m^2.7^)	30.4 (± 6.6)	26.2 (± 6.2)	0.01
Mean 24-h ABPM blood pressure	113/68		
Mean daytime ABPM blood pressure	117/72		
Mean nocturnal ABPM blood pressure	104/59		

*pSBP* peripheral systolic blood pressure, *pDBP* peripheral diastolic blood pressure, *cSBP* central systolic blood pressure, *cDBP* central diastolic blood pressure, *PP* pulse pressure, *PWVcf* carotid-femoral pulse wave velocity, *LVMI* left ventricular mass index

Twenty-four-hour ABPM data was available for 37 children with ADPKD (10 children had inadequate ABPM, were unable to tolerate or were too young to have ABPM). ABPM results are in Table 3. Thirteen of 37 children (35%) showed a lack of appropriate nocturnal dipping on 24-h ABPM (< 10% dip in SBP).

### Cardiovascular assessments

We found no significant difference in PWVcf between the children with ADPKD and the healthy children [mean 5.74 ± 1 m/s vs. 5.57 ± 0.9 m/s, *p* = 0.46, mean *Z*-score 1.21 vs. 0.78, *p* = 0.07]. There was a significantly higher LVMI in those with ADPKD compared to healthy children [mean 30.4 ± 6.6 g/m^2.7^ vs. 26.2 ± 6.2 g/m^2.7^, *p* = 0.01]. Although this was demonstrated on a study level, no children met the definition for left ventricular hypertrophy.

## Discussion

This is the first study to systematically assess blood pressure and cardiovascular status in a large population of children with ADPKD and compare with age-matched healthy controls. We have identified that those with ADPKD have higher mean peripheral and central blood pressures, compared to healthy children. Twenty-one percent of children in this study were hypertensive or on anti-hypertensive medication, which is consistent with ranges in the published literature [4]. That 8 children in our study were on anti-hypertensive medication suggests that the true mean BP values may be even greater in the ADPKD group, had they not been on anti-hypertensives at the time of the study. We also found that children with ADPKD had significantly higher indexed LV mass, compared to healthy controls.

The results of our study are in agreement with other paediatric work reporting an increased central blood pressure in children and young adults with ADPKD [12]. Central blood pressure characteristics have been reported in one adult study of 18 individuals with ADPKD; this study found higher central systolic and diastolic BP in young adults with ADPKD [15]. As well as observing a raised cBP, we have also found a lower PP amplification ratio in children with ADPKD. A lower PP amplification ratio may be an important marker of vascular stiffness and future cardiovascular risk [22]. A consequence of its resultant haemodynamic effect would be that for a given pPP, an individual with lower PP amplification will have a higher cPP and therefore a higher left ventricular afterload. Our study may provide evidence of arterial stiffening in young children with ADPKD at a median age of 11 years, but data on the relationship between these cardiovascular assessments and arterial stiffening are lacking in the paediatric population. We found a significant positive correlation between the PP amplification ratio and age and a negative correlation between PP amplification ratio and eGFR. Both findings are difficult to explain. Our study was not designed to investigate this, so whilst it is interesting to note, we cannot draw conclusions on the basis of this.

There have only been two previous similar studies of vascular health including children with ADPKD. Nowak et al. considered vascular health in children and young adults with ADPKD, but this study only included 15 individuals with ADPKD, and they had a median age of 21 years with 7 participants being 16 years old or younger. In this study, they found a raised PWVcf in those with ADPKD, compared to healthy controls [12]. Karava et al. studied 21 children and young adults aged 7–19 years and found an increased PWV in 4 (19%) children, but they did not have a healthy control comparison group [13]. Our study used the same methods prospectively in those with ADPKD and healthy controls and found no significant difference in PWVcf between children with ADPKD and healthy controls.

Although we did find some evidence of arterial stiffening in those with ADPKD through a lower PP amplification ratio, the lack of a significant difference in PWVcf may be due to the younger mean age of children in our study, compared to the study by Nowak et al. [12]. However, there have been other studies in young adults with ADPKD where no difference in PWVcf has been found [15]. The reasons for these conflicting findings are not clear, but these are likely related to different techniques for measuring both BP and PWV [23]. It may also be that significant large arterial stiffening occurs variably during childhood and young adulthood, and therefore, widespread vascular changes are not seen until later adult life. Our study also found that 35% of children with ADPKD had a loss of nocturnal dipping on 24-h ABPM; this is relatively consistent (although slightly lower) compared to a large recently published study by Massella et al. [8].

Other important findings in our study include further evidence of target organ damage in children with ADPKD, potentially as a result of hypertension or the disease itself. We found a higher mean LVMI in those with ADPKD, compared to healthy children. Whilst this finding is not novel [3], it adds to the evidence base suggesting that early cardiovascular changes are seen in children with ADPKD. We also found that 42% of patients with ADPKD had evidence of microalbuminuria, with 9% having an ACR above 10 g/mol. We report higher levels of microalbuminuria than some other studies, but this may be due to our urine samples being collected in the clinic randomly; therefore, there may be an orthostatic effect in some children. The mechanisms of proteinuria in early ADPKD are not yet fully understood, but theories suggest that glomerular hyperfiltration and abnormal endocytosis in tubules may account for this in part. Microalbuminuria in children with ADPKD is not consistently correlated to disease severity [24]. Nevertheless, our finding is important because it provides further rationale for initial treatment of hypertension with the renin-angiotensin-aldosterone system (RAAS) blockade, in view of the well-established antiproteinuric effects.

Strengths of our study include the consecutive recruitment of a relatively large population of children with ADPKD. The consecutive recruitment may also explain why the prevalence of hypertension in our study is lower than some other studies which may have suffered from selection bias. We also used a strict definition for hypertension as per current European guidelines [16]. Additional strengths include a well-matched group of healthy controls. Many previous studies of children with ADPKD have also included young adults, making it difficult to separate out the findings relevant to younger children and adolescents. Our study included only those aged less than 18 years, so the results can be truly applicable to paediatric patients.

Our study has some limitations that need to be acknowledged. Firstly, like many studies in paediatric ADPKD, our study is likely to suffer from some tertiary centre bias. Therefore, we may to some extent over-estimate the prevalence of hypertension and proteinuria in this cohort. We also acknowledge that urine samples collected in a clinic setting may also over-estimate microalbuminuria due to an orthostatic effect, although we would not expect such a significant number if it was purely due to this. Whilst our study population includes some children on anti-hypertensive therapy or with reduced eGFR which can add complexity to the interpretation of cardiovascular assessments, these individuals do not significantly skew the results and they can be seen clearly in Fig. 1 where their impact on pSBP is depicted.

The proposed mechanisms of hypertension in ADPKD have been described previously in our introduction. Given that most children with ADPKD have a low cyst burden, it is likely that mechanisms other than cyst proliferation and expansion also contribute to the development of hypertension in this population. Previous studies have shown that children with a higher cyst load have a higher risk of hypertension [25]. However, given that activation of the RAAS is still likely to be a major contributor to hypertension in children with ADPKD, we support the use of ACE inhibitors and angiotensin receptor blockers as the first-line anti-hypertensive agent in this population.

In conclusion, we have found that children with ADPKD have higher blood pressure levels and evidence of early cardiovascular abnormalities, compared to healthy children. There is also a high prevalence of microalbuminuria in this population. Further research is required to understand the mechanisms of elevated BP in children with ADPKD and its influence on the evolution of target organ damage. We propose a large population-based study of children at risk of ADPKD to determine the prevalence of hypertension and cardiovascular disease in this cohort, as well as performing cardiac and renal MRI to determine further markers of cardiovascular status and link these with cyst burden and renal size.

## References

[1] Cadnapaphornchai MA (2015). Autosomal dominant polycystic kidney disease in children. Curr Opin Pediatr.

[2] Ivy DD, Shaffer EM, Johnson AM, Kimberling WJ, Dobin A, Gabow PA (1995). Cardiovascular abnormalities in children with autosomal dominant polycystic kidney disease. J Am Soc Nephrol.

[3] Cadnapaphornchai MA, McFann K, Strain JD, Masoumi A, Schrier RW (2008). Increased left ventricular mass in children with autosomal dominant polycystic kidney disease and borderline hypertension. Kidney Int.

[4] Marlais M, Cuthell O, Langan D, Dudley J, Sinha MD, Winyard PJD (2016). Hypertension in autosomal dominant polycystic kidney disease: a meta-analysis. Arch Dis Child.

[5] S. Polubothu, A. Richardson, L. Kerecuk, and M. Sinha, Autosomal dominant polycystic kidney disease in children., *BMJ*, vol. 353, no. June, p. i2957, Jun. 2016 10.1136/bmj.i2957 27266528

[6] De Rechter S, Breysem L, Mekahli D (2017). Is autosomal dominant polycystic kidney disease becoming a pediatric disorder?. Front Pediatr.

[7] Dudley J (2019). Clinical practice guideline monitoring children and young people with, or at risk of developing autosomal dominant polycystic kidney disease (ADPKD). BMC Nephrol.

[8] Massella L (2018). Prevalence of hypertension in children with early-stage ADPKD. Clin J Am Soc Nephrol.

[9] Schrier RW, Johnson AM, McFann K, Chapman AB (2003). The role of parental hypertension in the frequency and age of diagnosis of hypertension in offspring with autosomal-dominant polycystic kidney disease. Kidney Int.

[10] A. Cadnapaphornchai M (2013). Hypertension in children with autosomal dominant polycystic kidney disease (ADPKD). Curr Hypertens Rev.

[11] Schrier RW (2014). Blood pressure in early autosomal dominant polycystic kidney disease. N Engl J Med.

[12] Nowak KL, Farmer H, Cadnapaphornchai MA, Gitomer B, Chonchol M (2017). Vascular dysfunction in children and young adults with autosomal dominant polycystic kidney disease. Nephrol Dial Transplant.

[13] Karava V, Benzouid C, Hogan J, Dossier C, Denjean AP, Deschênes G (2018) Early cardiovascular manifestations in children and adolescents with autosomal dominant polycystic kidney disease: a single center study. Pediatr Nephrol:4–6 10.1007/s00467-018-3964-9 29774463

[14] Agabiti-Rosei E (2007). Central blood pressure measurements and antihypertensive therapy: a consensus document. Hypertension.

[15] Borresen ML, Wang D, Strandgaard S (2007). Pulse wave reflection is amplified in normotensive patients with autosomal-dominant polycystic kidney disease and normal renal function. Am J Nephrol.

[16] Lurbe E (2016). 2016 European Society of Hypertension guidelines for the management of high blood pressure in children and adolescents. J Hypertens.

[17] Schwartz GJ, Haycock GB, Edelmann CM, Spitzer A (1976). A simple estimate of glomerular filtration rate in children derived from body length and plasma creatinine. Pediatrics.

[18] Schwartz GJ (2009). New equations to estimate GFR in children with CKD. J Am Soc Nephrol.

[19] National High Blood Pressure Education Program Working Group on High Blood Pressure in Children and and Adolescents (2004). The fourth report on the diagnosis, evaluation, and treatment of high blood pressure in children and adolescents. Pediatrics.

[20] Sinha MD, Booth CJ, Reid CJD (2011). Factors affecting success of blood pressure measurements during ambulatory blood pressure monitoring in children with renal disease. Cardiol Young.

[21] Reusz GS (2010). Reference values of pulse wave velocity in healthy children and teenagers. Hypertension.

[22] Avolio AP (2009). Role of pulse pressure amplification in arterial hypertension: experts’ opinion and review of the data. Hypertension.

[23] Keehn L, Milne L, McNeill K, Chowienczyk P, Sinha MD (2014). Measurement of pulse wave velocity in children: comparison of volumetric and tonometric sensors, brachial-femoral and carotid-femoral pathways. J Hypertens.

[24] Schrier RW (2014). Predictors of autosomal dominant polycystic kidney disease progression. J Am Soc Nephrol.

[25] Cadnapaphornchai MA, McFann K, Strain JD, Masoumi A, Schrier RW (2009). Prospective change in renal volume and function in children with ADPKD. Clin J Am Soc Nephrol.

